# Decoding the proteomic changes involved in the biofilm formation of *Enterococcus faecalis* SK460 to elucidate potential biofilm determinants

**DOI:** 10.1186/s12866-019-1527-2

**Published:** 2019-06-28

**Authors:** Karthika Suryaletha, Lekshmi Narendrakumar, Joby John, Megha Periyappilly Radhakrishnan, Sanil George, Sabu Thomas

**Affiliations:** 1 Cholera and Biofilm Research Laboratory, Pathogen Biology, Rajiv Gandhi Centre for Biotechnology, (National Institute under the Department of Biotechnology, Government of India), Trivandrum, Kerala 695014 India; 2 Department of Surgery, Government Medical College Hospital, Trivandrum, Kerala 695011 India; 3 Interdisciplinary Biology, Rajiv Gandhi Centre for Biotechnology, Trivandrum, Kerala 695014 India

**Keywords:** *Enterococcus faecalis*, Biofilm determinants, Quantitative proteomics, Metabolic pathways, Stress response, *luxS*

## Abstract

**Background:**

*Enterococcus faecalis* is a major clinically relevant nosocomial bacterial pathogen frequently isolated from polymicrobial infections. The biofilm forming ability of *E. faecalis* attributes a key role in its virulence and drug resistance. Biofilm cells are phenotypically and metabolically different from their planktonic counterparts and many aspects involved in *E. faecalis* biofilm formation are yet to be elucidated. The strain *E. faecalis* SK460 used in the present study is *esp* (Enterococcal surface protein) and *fsr* (two-component signal transduction system) negative non-gelatinase producing strong biofilm former isolated from a chronic diabetic foot ulcer patient. We executed a label-free quantitative proteomic approach to elucidate the differential protein expression pattern at planktonic and biofilm stages of SK460 to come up with potential determinants associated with Enterococcal biofilm formation.

**Results:**

The Gene Ontology and Kyoto Encyclopedia of Genes and Genomes (KEGG) enrichment analyses of proteomic data revealed that biofilm cells expressed higher levels of proteins which are associated with glycolysis, amino acid biosynthesis, biosynthesis of secondary metabolites, microbial metabolism in diverse environments and stress response factors. Besides these basic survival pathways, LuxS-mediated quorum sensing, arginine metabolism, rhamnose biosynthesis, pheromone and adhesion associated proteins were found to be upregulated during the biofilm transit from planktonic stages. The selected subsets were validated by quantitative real-time PCR. In silico functional interaction analysis revealed that the genes involved in upregulated pathways pose a close molecular interaction thereby coordinating the regulatory network to thrive as a biofilm community.

**Conclusions:**

The present study describes the first report of the quantitative proteome analysis of an *esp* and *fsr* negative non gelatinase producing *E. faecalis*. Proteome analysis evidenced enhanced expression of glycolytic pathways, stress response factors, LuxS quorum signaling system, rhamnopolysaccharide synthesis and pheromone associated proteins in biofilm phenotype. We also pointed out the relevance of LuxS quorum sensing and pheromone associated proteins in the biofilm development of *E. faecalis* which lacks the Fsr quorum signaling system. These validated biofilm determinants can act as potential inhibiting targets in Enterococcal infections.

**Electronic supplementary material:**

The online version of this article (10.1186/s12866-019-1527-2) contains supplementary material, which is available to authorized users.

## Background

Enterococci have emerged as a global cause of nosocomial infection and are frequently associated with chronic ulcers, urinary tract infections, endocarditis and indwelling medical device-related infections. The robust biofilm formation and inherent multidrug resistance in Enterococci have uplifted it as a challenging nosocomial pathogen. Biofilms are cell populations irreversibly attached to surfaces and encased within a self-produced hydrated matrix of exopolymeric substances, proteins, polysaccharides, extracellular DNA (eDNA) and water channels. Biofilm confers high antibiotic tolerance ability and resistance to phagocytosis and hence play a prime role in critical colonization, establishment and chronicity of infections. This renders Enterococci to be recalcitrant towards the current treatment strategies. Owing to its egressing clinical significance, the pace of enterococcal biofilm research has accelerated in the past few years for providing a better understanding of intricate underlying mechanisms of biofilm formation.

In *E.faecalis*, fsr two-component signal transduction system is a well-defined quorum sensing system which regulates biofilm formation by gelatinase production [1]. Previous studies on enterococcal biofilm have suggested several other biofilm associated factors including enterococcal surface protein (*esp*) [2], aggregation substance [3], pili [4], autolysin [5] and D-alanylation of Lipoteichoic acid (dltABCD) [6]. Several studies on *E. faecalis* comparing biofilm formers and non-biofilm formers have identified protein translation machinery, aromatic amino acid biosynthesis and sugar and sulfate permease transporter systems to have a momentous role in biofilm formation [7, 8]. *E. faecalis* SK460 used in the present study is isolated from a chronic diabetic ulcer patient [9] and is devoid of several well-defined biofilm associated factors including fsr quorum signaling, gelatinase production and enterococcal surface protein. Lack of these biofilm determinants does not affect the biofilm forming potential of SK460. This led us to focus on the role of differential protein expression pattern in biofilm phenotype of this strong biofilm former. The present study utilized label-free quantitative approach to decipher the protein expression pattern of *E. faecalis* SK460 at planktonic and biofilm stages to elucidate the unexplored links in understanding the enterococcal biofilms. This helps to deliver the comprehensive knowledge regarding the metabolic pathways and cellular processes involved in Enterococcal biofilm to come up with potential biofilm inhibiting targets.

## Results

### Biofilm forming potential of *E. faecalis* SK460

**Fig. 1:**
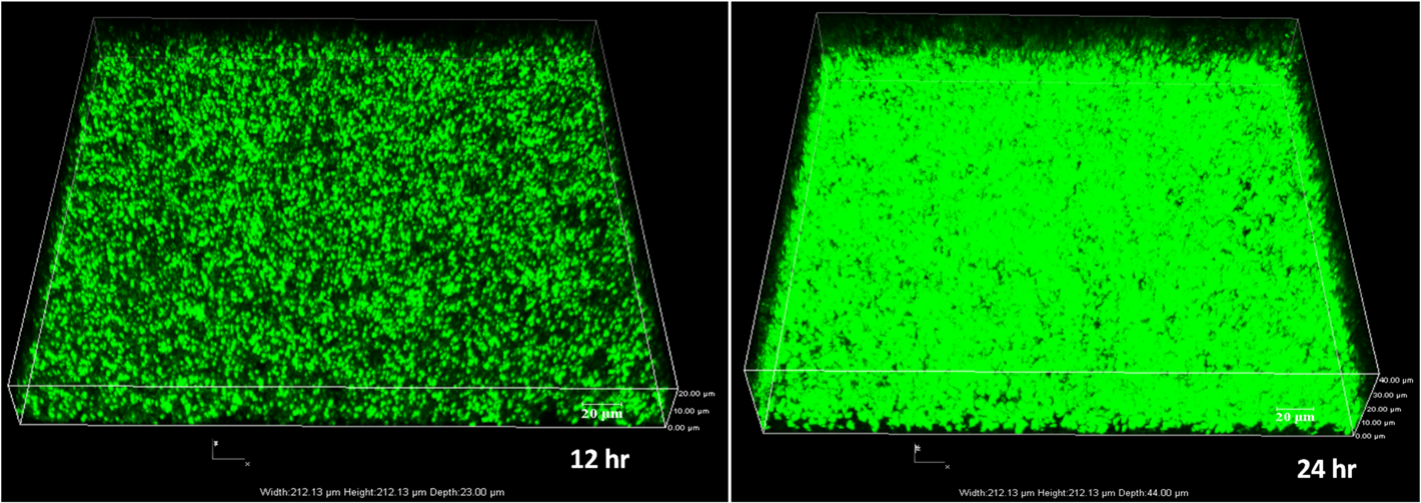
Confocal laser scanning microscopy images of biofilm formation of *E. faecalis* SK460 at 12 h and 24 h. The images are processed using NIS-Element AR software, version 4.00.04

Confocal Laser Scanning Microscopy analysis evidenced the high biofilm forming ability of *E. faecalis* SK460 (Fig. 1). The 24 h old biofilm showed an average thickness of approximately 40 μm.

### Proteome profile obtained in planktonic and biofilm stages

Label-free quantitative proteomics identified 657 proteins from planktonic stages and 553 proteins from biofilm stages. Of these, 233 (29.6%) and 129 (16.4%) proteins were identified exclusively in the planktonic and biofilm stages respectively and 424 (53.9%) proteins were detected at both stages.

### Physico-chemical properties of identified proteome

The hydrophobic nature of the identified proteins was calculated using the GRAVY (grand average hydropathy) tool and the score obtained ranges between − 1.6 and 1 (Additional file 2: Figure S1a). Nearly 88% of the proteins were hydrophilic (< 0) and the rest were hydrophobic or membranous (> 0) in nature. The compute pI/MW tool revealed that the extracted proteins were within the pI range of 3.5–11.5 and molecular weight of 4–186 kDa (Additional file 3: Figure S1b). 90% of the proteins were within the range of 80 kDa.

### Functional categorization of proteome at planktonic and biofilm stages

#### KEGG pathways

**Fig. 2:**
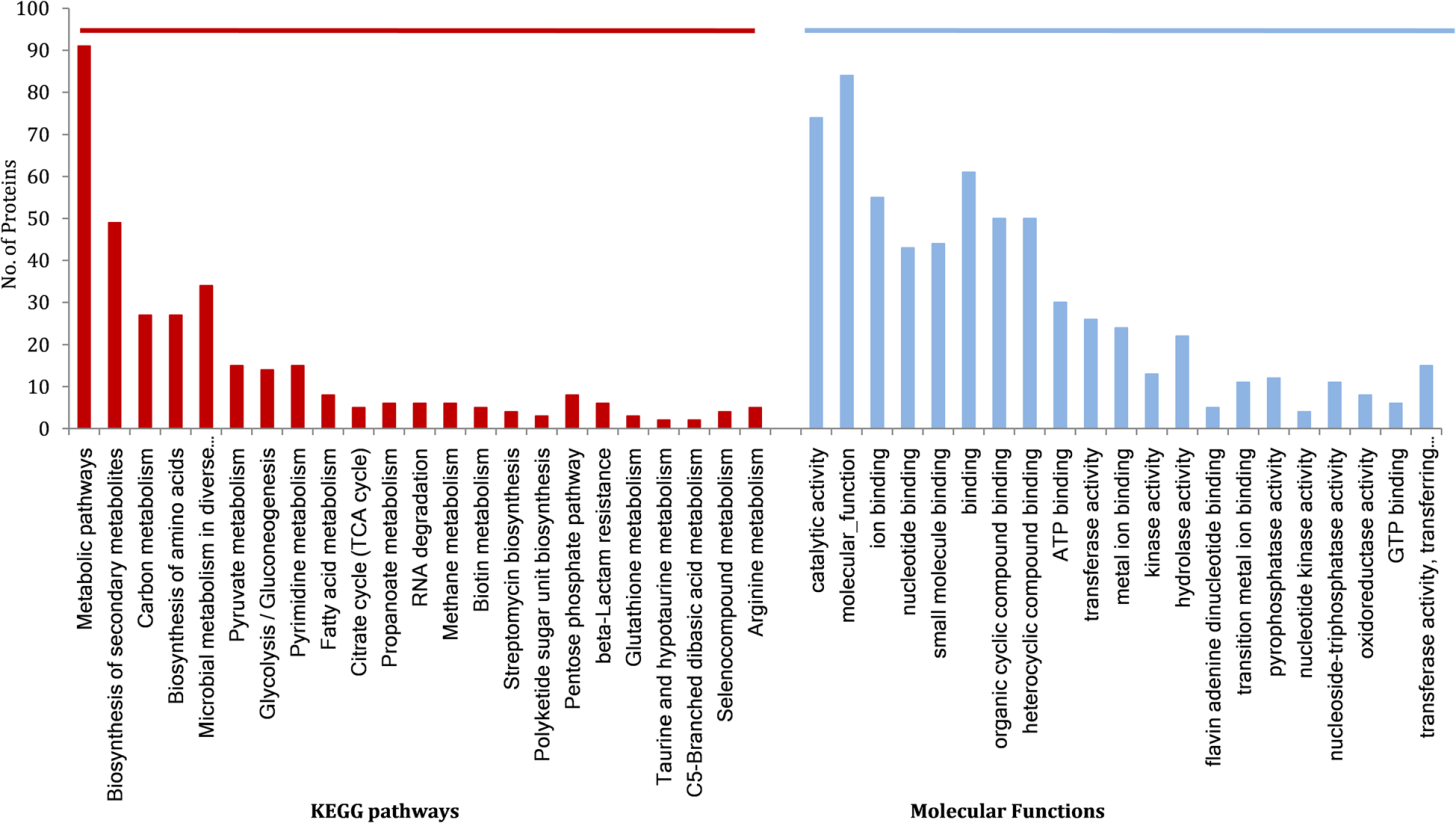
KEGG pathways and molecular functions assigned to categorize the upregulated proteins obtained in biofilm stages of *E. faecalis* SK460

The proteins identified in the planktonic and biofilm stages were assigned to define KEGG pathways. The major pathways were associated with ribosome (72 hits), pyruvate metabolism (27 hits), pyrimidine metabolism (50 hits), amino acid biosynthesis (69 hits) and antibiotics (122 hits), Fatty acid metabolism (15 hits), RNA degradation (13 hits), biosynthesis of secondary metabolites (157 hits), glycolysis/gluconeogenesis (40 hits), citrate cycle (TCA cycle) (7 hits), microbial metabolism in diverse environments (110 hits), propanoate metabolism (11 hits) and butanoate metabolism (9 hits). Furthermore, the proteins upregulated/unique to biofilm stages were assigned to KEGG pathways and revealed the enrichment of glycolysis/gluconeogenesis, pyruvate metabolism, biosynthesis of secondary metabolites, microbial metabolism in diverse environments and arginine metabolism (Fig. 2). More than 50% of the total proteins involved in these pathways were detected to be upregulated in biofilm stages of SK460.

### Biological processes

The proteins were clustered based on gene ontology (GO) analysis and its relative expression at planktonic and biofilm stages were analyzed. Comparative proteome analysis revealed that several ribosomal proteins and proteins involved in DNA replication and transcription were found to be normally expressed or slightly upregulated. The relative abundance of some proteins involved in nucleotide metabolism and protein biosynthesis was affected to differing degrees under biofilm conditions. Major functional categories of proteins upregulated in biofilm stages are listed in Table 1.

**Table 1:** List of proteins enhanced in biofilm stages of *E. faecalis* SK460

NCBI-ID	Pathways/cellular processes
Amino acid metabolism	
NP_816782.1	Succinyl-diaminopimelate desuccinylase (EF_3178)
NP_816328.1	MTA/SAH nucleosidase (*pfs*)
NP_815300.1	Cysteine synthase A (*cysK*)
NP_815281.1	Chorismate synthase (*aroC*)
NP_815821.1	Glutamine synthetase, type I (*glnA*)
NP_814529.1	S-adenosylmethionine synthetase (*metK*)
NP_815283.1	3-phosphoshikimate 1-carboxyvinyltransferase (*aroA*)
NP_816193.1	Serine hydroxymethyltransferase (*glyA*)
NP_816069.1	Threonine synthase (*thrC*)
NP_815279.1	3-deoxy-7-phosphoheptulonate synthase (EF_1562)
NP_816700.1	Bifunctional glutamate--cysteine ligase/glutathione synthetase (*gshF*)
NP_816650.1	Glutamyl-aminopeptidase (*pepA*)
NP_814940.1	Acetolactate synthase (EF_1213)
NP_815294.1	Dihydrofolate reductase (*folA*)
Glycolysis, citric acid cycle, pentose phosphate pathway	
NP_815638.1	Triosephosphate isomerase (*tpiA*)
NP_815639.1	Phosphoglycerate kinase (*pgk*)
NP_814897.1	Fructose-bisphosphate aldolase (*fba*)
NP_813994.1	Phosphoglycerate mutase (*gpmA*)
NP_815245.1	Glyceraldehyde 3-phosphate dehydrogenase (*gap-*1)
NP_815637.1	Enolase (*eno*)
NP_815137.1	Glucose-6-phosphate isomerase (*pgi*)
NP_815075.1	Pyruvate dehydrogenase complex, E1 component subunit beta (*pdhB*)
NP_815076.1	Dihydrolipoamide acetyltransferase (*aceF*)
NP_816104.1	Pyruvate carboxylase (*pycA*)
NP_813976.1	Deoxyribose-phosphate aldolase (*deoC*)
NP_813986.1	Phosphopentomutase (*deoB*)
NP_814782.1	6-phosphogluconate dehydrogenase (*gnd*)
Stress response, chaperones and DNA repair	
NP_814237.1	OsmC/Ohr family protein (EF_0453)
NP_816831.1	Dps family protein (EF_3233)
NP_816649.1	Thioredoxin family protein (EF_3036)
NP_814938.1	NADH peroxidase (*npr*)
NP_815447.1	General stress protein (EF_1744)
NP_816369.1	Alkyl hydroperoxide reductase subunit C (*ahpC*)
NP_813885.1	gls24 protein
NP_816648.1	Universal stress protein (EF_3035)
NP_816867.1	Glutathione reductase (*gor*)
NP_816700.1	Bifunctional glutamate--cysteine ligase/glutathione synthetase (*gshF*)
NP_816368.1	Thioredoxin reductase/glutathione-like protein (EF_2738)
NP_814049.1	L-lactate dehydrogenase (*ldh1*)
NP_816550.1	AhpC/TSA family protein (EF_2932)
NP_815246.2	GTPase ObgE
NP_815059.1	Thioredoxin reductase (*trxB*)
NP_815353.1	Transcriptional repressor CodY (*codY*)
NP_816010.1	ATP-dependent Clp protease, ATP-binding protein ClpB (*clpB*)
NP_814518.1	ATP-dependent Clp protease proteolytic subunit (*clpP*)
NP_816516.1	Cyclophilin type peptidyl-prolyl cis-trans isomerase (EF_2898)
NP_814457.1	ATP-dependent Clp protease, ATP-binding protein ClpE (*clpE*)
NP_816532.1	Transcription elongation factor GreA
NP_815030.1	dnak protein
NP_815032.1	dnaJ protein
NP_815029.1	Heat shock protein GrpE (*grpE*)
NP_814465.1	Trigger factor (TIG)
NP_816273.1	GroS (10 kDa chaperonin)
NP_816272.1	GroL(60 kDa chaperonin)
NP_814060.1	Fn1610 (33 kDa chaperonin) (*hslO*)
NP_814686.1	Uracil-DNA glycosylase (*ung*)
NP_814720.1	DNA repair protein RecN (*recN*)
Rhamnose biosynthesis	
NP_815854.1	dTDP-glucose 4,6-dehydratase (*rfbB*)
NP_815853.1	dTDP-4-dehydrorhamnose reductase (EF_2191)
NP_815856.1	Glucose-1-phosphate thymidylyltransferase (*rfbA*)
Arginine metabolism	
NP_813908.1	Ornithine carbamoyltransferase (*arcB*)
NP_815423.1	Carbamoyl phosphate synthase small subunit (*carA*)
NP_814373.1	Ornithine cyclodeaminase (EF_0616)
NP_813909.1	Carbamate kinase (*arcC1*)
NP_813907.1	Arginine deiminase (*arcA*)
NP_814719.1	ArgR family transcriptional regulator (EF_0983)
Quorum sensing and pheromone associated lipoprotein	
NP_814911.1	S-ribosylhomocysteinase (*luxS*)
NP_816853.1	Pheromone cAD1 lipoprotein (EF_3256)
NP_814793.1	Pheromone binding protein (EF_1060)
NP_815061.1	Pheromone cAM373 lipoprotein (EF_1340)
Adhesion associated proteins	
NP_814266.1	Aggregation substance (EF_0485)
NP_814342.1	Adhesion lipoprotein (EF_0577)
NP_814975.1	Fibronectin/fibrinogen binding protein (EF_1249)
Autolysin and D-Alanylation of teichoic acid	
NP_814543.1	Autolysin (EF_0799)
NP_816376.1	D-alanine--poly(phosphoribitol) ligase subunit 2 (*dltC*)
NP_816375.1	dltD protein (*dltD*)

In general, major glycolytic enzymes and several enzymes in the citric acid cycle and pentose phosphate pathway were found to be in higher abundance in biofilm stages of SK460. Enzymes involved in rhamnose biosynthesis which forms the part of enterococcal polysaccharide antigen gene cluster was also found to be upregulated in biofilm stages. Compared to planktonic forms, Enterococcal biofilms showed a hike in stress response factors, DNA repair system, molecular chaperones, pheromone associated lipoproteins and other adhesion associated proteins. Besides, the present proteome analysis established the role of *luxS* mediated quorum sensing in enterococcal biofilm development.

**Fig. 3:**
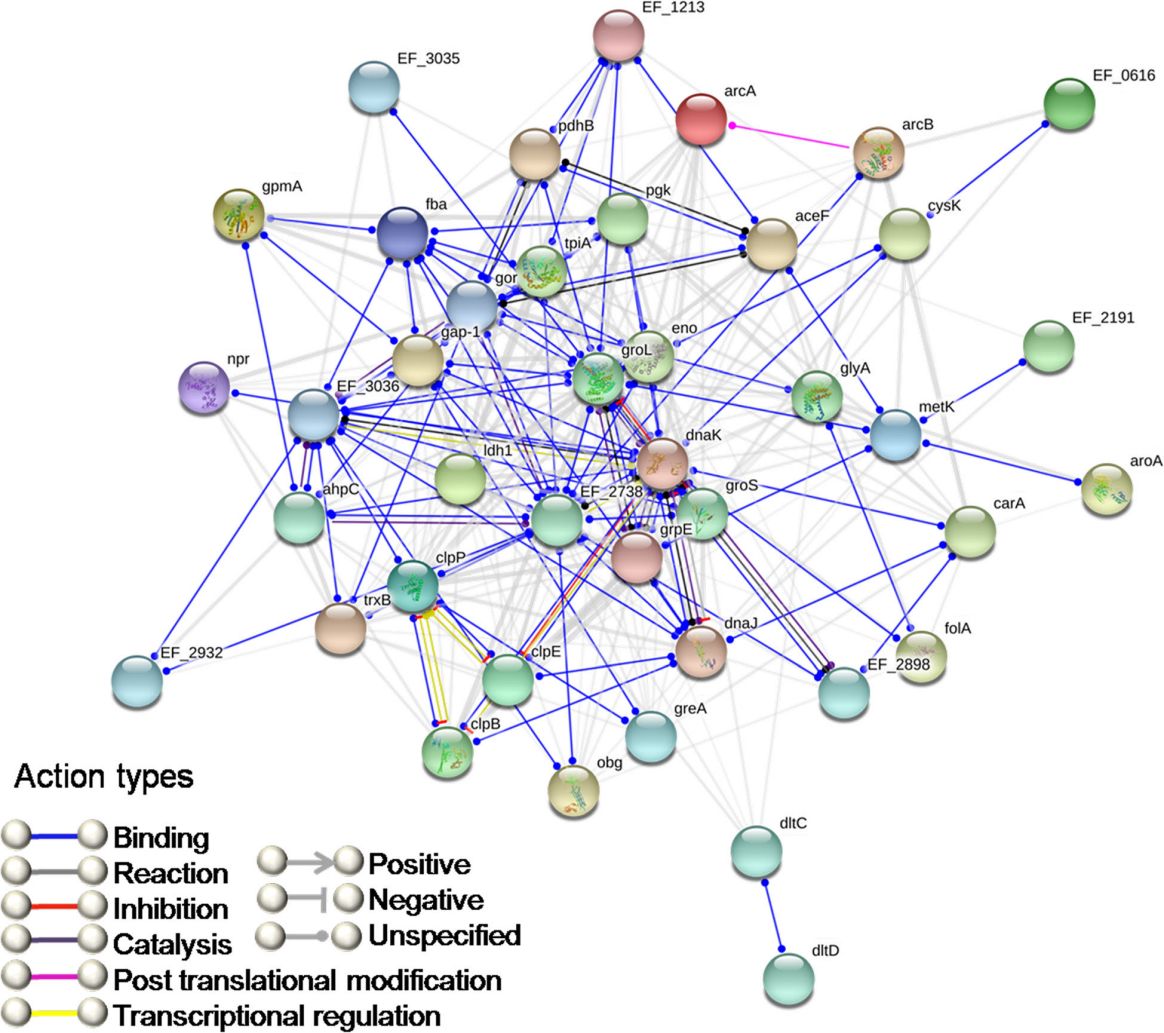
STRING analysis (Version 10.5) showing the predicted molecular action among the upregulated proteins in biofilm stages. The gene nomenclatures are depicted in Table 1. Colored nodes: query proteins and first shell of interactors. Empty nodes: proteins of unknown 3D structure. Filled nodes: 3D structure is known/predicted. Edges represent protein-protein associations

STRING analysis predicted the molecular interactions among the highly expressed proteins in biofilm stages with enrichment *p*-value < 1.0e-16 and is shown in Fig. 3. The functional interactome of the whole upregulated protein sets is depicted in Additional file 2: Figure S2.

### Validation of selected biofilm associated genes by RT-PCR

**Fig. 4:**
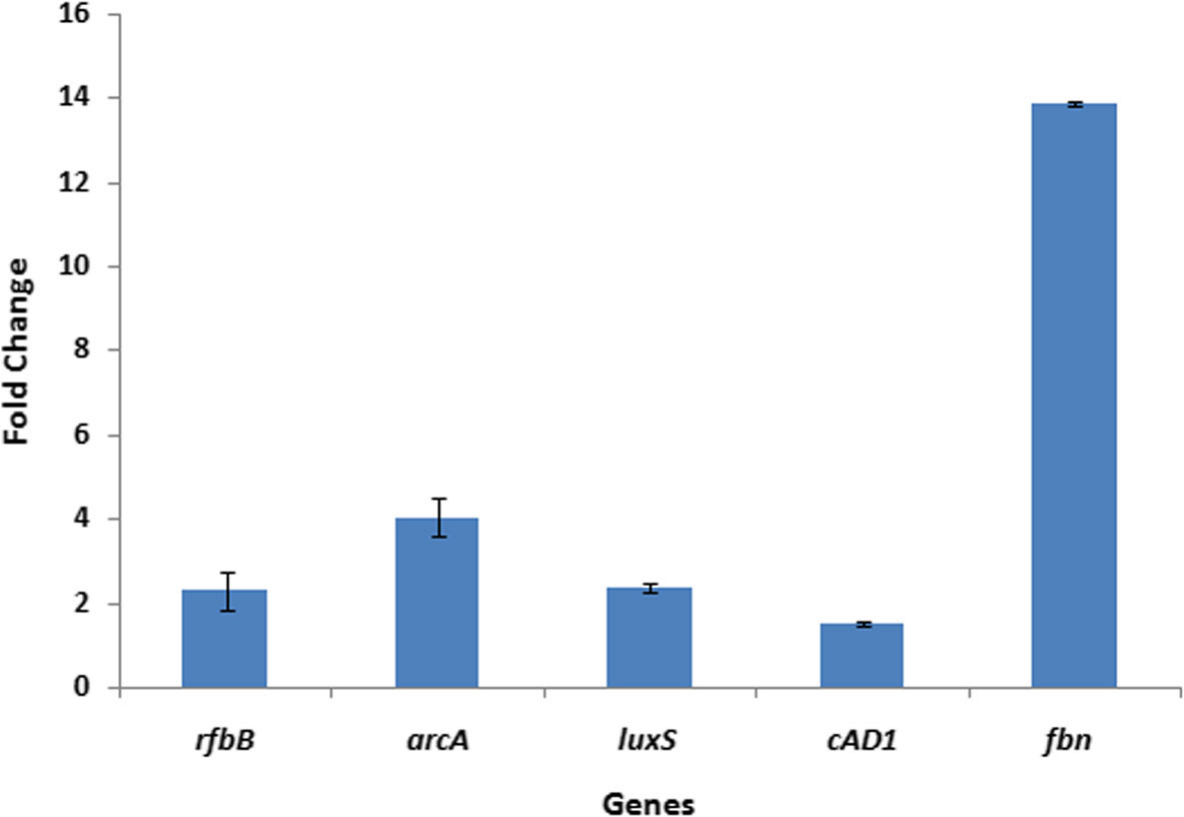
Real time qPCR validation of selected biofilm associated genes. Fold change expression of genes observed in biofilm stage with respect to its corresponding planktonic stage

The genes *rfbB* (Rhamnose biosynthesis), *arcA* (Arginine deiminase), *luxS* (Quorum sensing), *cAD1* (Pheromone cAD1 lipoprotein) and *fbn* (Fibrinogen/Fibronectin binding protein associated with adhesion) were selected to monitor the fold change expression in biofilm stage when compared to planktonic stage and the results are shown in Fig. 4. All these five genes were found to be upregulated and adhesion protein (*fbn*) showed a comparatively large fold increase in expression. This substantiates the significant role of these pathways in the development of mature biofilm.

## Discussion

*E. faecalis* is a clinically relevant hospital associated pathogen and the recalcitrant enterococcal biofilms are known to play a foremost role in establishing chronic infections. The wound isolate *E. faecalis* SK460 was proved to be a robust biofilm former by confocal laser scanning microscopy. Though SK460 is a potent biofilm former, it is devoid of several well-known biofilm determinants including fsr quorum signaling system and gelatinase production. Previous studies have established the role of fsr regulated gelatinase production and enterococcal surface protein in Enterococcal biofilm development. Besides these aforementioned determinants, several other factors are yet to be elucidated to pick out the unidentified factors associated with enterococcal biofilm. Hence, our prime focus was to decipher the metabolic pathways and physiological processes related to the Enterococcal biofilm development utilizing the label-free quantitative proteomic approach.

In the present investigation, the majority of proteins involved in basic survival mechanisms including DNA replication and transcription were either normally expressed or slightly upregulated in biofilm stages. Starting from the planktonic form to microcolonies and maturing biofilm, an intracellular pool of carbohydrates, amino acids and lipids is required to facilitate the development of biomass required for the sessile growth period. Besides these, certain putative transcriptional regulators and DNA binding response regulators have also shown an increasing trend in biofilm stages. The differential expression of major metabolic pathways and cellular processes upregulated in biofilm stages are discussed in this section.

### Pathways upregulated in biofilm stages

#### Amino acid metabolism

In the present investigation, various enzymes including chorismate synthase (3.97 fold), 3-phosphoshikimate 1-carboxyvinyltransferase (1.5 fold), and 3-deoxy-7-phosphoheptulonate synthase (1.56 fold) which are involved in the biosynthesis of aromatic amino acids were found to be upregulated in biofilm stages. A similar trend of enhanced expression of aromatic amino acid biosynthesis enzymes has already been reported in a previous proteomic study performed on strong and weak biofilm forming *E. faecalis* strains [8]. Other major amino acid biosynthesis enzymes upregulated were Succinyl-diaminopimelate desuccinylase (6.35 fold), MTA/SAH nucleosidase (2.88 fold), cysteine synthase A (3.387 fold), Glutamine synthetase (5.81 fold), Acetolactate synthase (1.89 fold), etc. Previous studies on several other bacterial genera also revealed the role of amino acids in the formation of tight microcolonies and in maintaining the stability of mature biofilm [10–12]. Hence it is evident that during the biofilm maturation process, a series of amino acids and its related metabolites will be upregulated for contributing to extracellular matrix component of robust biofilms.

#### Carbohydrate metabolism

The present study evidenced the augmented production of seven major glycolytic enzymes (2.5 to 9 fold change) in biofilm stages compared to planktonic forms as listed in Table 1. Previous studies have shown the significant role of Glyceraldehyde 3-phosphate dehydrogenase in *P. aeruginosa* and *Staphylococcus xylosus* biofilms [13, 14] and also an upregulated expression in microaerophilic conditions in *E.coli* [15]. It is assumed that the cells within the biofilm will be in microaerophilic condition and needs a high level expression of these enzymes for adapting in the oxygen-limited environment. Another glycolytic enzyme phosphoglycerate mutase was found to be upregulated in *S.xylosus* biofilm [16] and is said to have a bifunctional role which helps in the synthesis of various EPSs, thus playing a significant role in the synthesis of core biofilm matrix [17]. Thus the glycolytic pathway is found to be one of the essential factors among biofilm survival mechanisms. Pyruvate dehydrogenase complex and several enzymes involved in Tricarboxylic acid (TCA) cycle and pentose phosphate pathways were also observed to be enhanced during the biofilm formation of SK460.

### Stress response factors and protein folding

Several proteins involved in stress response including universal stress protein (10.69 fold), and General stress protein (3.42 fold) were identified to be steadily increased in biofilm stages. Similarly, universal stress proteins were found to be upregulated in *Porphyromonas gingivalis* and *Sulfolobus solfataricus* biofilms and showed impaired biofilm upon inactivation of these genes [18, 19]. Signaling pathways for inducing stress response within the biofilm are cell-density dependent quorum sensing and the starvation–activated stringent response where the latter helps bacteria in adapting to nutrient deprivation. Nguyen et al. [20] identified the role of this stringent response in protecting bacteria within the biofilm from antimicrobial stress. It also activates the production of catalase and superoxide dismutase in stress condition. Enhanced expression of GTPase ObgE (1.53 fold) suggested the tendency of biofilm cells to attain the persistence nature in response to nutrient deprivation as suggested by previous findings [21]. Similarly, proteins like DPS family protein (5.41 fold), Thioredoxin family protein (4.75 fold), Alkyl hydroperoxide reductase (2.33 fold) and H_2_O_2_ scavenging proteins (9.11 fold) involved in reactive oxygen stress were also found to be over synthesized. It was also noticed that Gls24 is overexpressed by 9.2 fold in biofilm cells, suggesting its role in Enterococcal biofilm which is in accordance with the previous reports. Gls24 was found to be related to the stress and virulence of *E. faecalis* and was considered as a possible immunotherapy target [22].

Chaperonins and chaperones including GroL (1.63 fold), GroS (3.49 fold), several heat shock proteins, etc. were also found to be relatively abundant in SK460 biofilm. Enhanced levels of chaperones ensure the proper protein folding which enables *E. faecalis* to thrive within the biofilm. Clp proteases involved in the degradation of misfolded proteins also showed elevated expression in biofilm stages. Several studies have proved the role of chaperone DnaK in curli-dependent biofilm formation and are considered as a potential target for anti-biofilm compounds [23–25].

### Rhamnose biosynthesis

Rhamnose biosynthesis enzymes including glucose-1-phosphate thymidylyltransferase (RfbA), dTDP-glucose 4,6-dehydratase (RfbB) and dTDP-4-dehydrorhamnose reductase (RfbD) were found to be more than threefold enhanced expression in the biofilm stage. Rhamnose biosynthesis enzymes belong to Enterococcal polysaccharide antigen (epa) gene cluster and were proved to have a major role in virulence in mouse peritonitis model [26, 27] and intestinal colonization [28]. This rhamnopolysaccharide epa also confers protection against high salt concentration and are said to be involved in osmotic stress response in *E. faecalis* [29]. Epa also confers resistance to various stresses including those of oxidative stress, stress towards ethanol, bile acids, detergent SDS and antimicrobial peptides. Decreased EPS production and colonization was observed due to knockout of rhamnose biosynthesis in *Azospirillum brasilense* [30]. Thus rhamnopolysaccharide serves as a major extracellular matrix component as well as enables the cells to survive within the stressed environment of the matrix.

### Arginine metabolism

Arginine deiminase (ArcA), ornithine carbamoyltransferase (ArcB) and carbamate kinase (ArcC) are enzymes involved in arginine deiminase (ADI) pathway and were found to be highly expressed (ranging from 1.9 to 8 fold) in *E. faecalis* biofilm in the present study. Ammonia synthesis via the ADI pathway is important to reduce the pH stress within the microcolonies. Mutants of arginine deiminase have showed decreased viability in *S. epidermidis* biofilm, and ADI is proved to have a role in pH homeostasis in biofilms [31]. Transcription of arc operon is often arginine dependent via ArgR family of regulators (ArgR and AhrC) which were found to be active in biofilm stages. ADI pathway is generally induced under anaerobic conditions and hence this may have a role in the oxygen reduced environment within the biofilm.

### Quorum sensing (QS) and pheromone associated proteins

S-ribosylhomocysteinase (LuxS) was shown to have a rising trend with more than threefold increased expression in biofilm stages. Previous studies evidenced that *luxS* mutants developed an altered biofilm and exhibited enhanced cell-surface hydrophobicity in *E. faecalis* [32]. Several other studies also had a similar observation of the negative impact of *luxS* mutants in *Streptococcus anginosus*, and *S. mutans* biofilms [33, 34]. But still, the role of *luxS* dependent quorum sensing is not well established in *E. faecalis* biofilm.

Pheromone cAD1 lipoprotein (1.91 fold) and other pheromone associated proteins were upregulated in biofilm stages. Surface-associated pheromone binding lipoproteins act as essential components for adhesion, colonization and virulence and are also involved in stress response. Varahan et al. [35] showed that pheromone transporter mutants displayed altered biofilm architecture with a significant reduction in biofilm biomass compared to wild type suggesting the role of pheromones and associated proteins in biofilm development.

### Adhesion associated proteins

Aggregation substance was already proved to have a role in Enterococcal biofilm formation [3, 36]. Adhesion lipoproteins are surface-associated lipoproteins involved in Enterococcal adherence, colonization and virulence [37] and are directly linked to biofilm formation as in agreement with our findings. Fibronectin/fibrinogen binding proteins also showed more than tenfold increase in biofilm stages and were suggested to have a significant role in *E. faecalis* associated urinary tract infections in a murine model [38] and endocarditis in rats [39]. Fibronectin binding protein mutants of community-associated MRSA losses the ability for fibronectin binding thereby preventing the establishment of biofilm in host tissue [40]. These proteins were assumed to be associated with bacterial aggregation and involve in primary attachment to a surface and hence can be considered as a suitable biofilm inhibiting target.

### Other major factors

Several DNA binding response regulators, phosphotransferase system transporter proteins, Autolysin, D-alanylation of lipoteichoic acid (dltABCD), etc. were also found to be more than two-fold upregulated in biofilm stages. Of these, autolysin is well known to be associated with the eDNA release in the matrix and structural integrity of the biofilm. Previous report showed that autolysin deficient mutants had defects in primary attachment and eDNA release which are mainly associated with the accumulative phase for maturation and structural stability of biofilm in *E. faecalis* [5]. These mutants lack DNase1-sensitive fibrous network which is found to have a role in biofilm stability. D-alanylation of lipoteichoic acid (*dltC* and *dltD*) was also found to be abundant in biofilm mode (2.36 to 4.17 fold), possibly incorporating lipoteichoic acid into the EPS matrix. Lack of d-alanine esters results in a stronger negative net charge on the bacterial cell surface and *dlt* mutants showed a reduction in biofilm formation on polystyrene surfaces. Furthermore, dltABCD operon is involved in the pathogenesis of *E. faecalis* leading to enhanced biofilm formation, host tissue attachment and increased resistance to antimicrobial peptides [6].

STRING analysis revealed that upregulated genes including stress response factors, major glycolytic enzymes, arginine metabolism and rhamnose biosynthesis were either directly or indirectly poses a close molecular interaction (*p*-value < 1.0e-16), thereby regulating each other in stressful environment prevailing during biofilm development. Among the upregulated cellular processes, *rfbB* (Rhamnose biosynthesis), *arcA* (Arginine deiminase), *luxS* (Quorum sensing), *cAD1* (Pheromone cAD1 lipoprotein) and *fbn* (Fibrinogen/Fibronectin binding protein associated with adhesion) were selected for gene expression analysis and were found to be enhanced by 2.29, 4.03, 2.35, 1.5 and 13.87 fold respectively in biofilm stages. Of these, *luxS* mediated quorum sensing system is attributed to play a major role in *E. faecalis* SK460 biofilm which is devoid of fsr two-component signal transduction system. Accordance of the proteome data with RT-PCR results confirms the reliability of the analysis, serving as a validation for the identified determinants of Enterococcal biofilm development.

## Conclusion

In *E. faecalis*, comparative proteome analysis revealed that pathways belonging to carbohydrate metabolism including glycolysis and pyruvate metabolism, amino acid metabolism, biosynthesis of secondary metabolites and microbial metabolism in diverse environments comprising stress response factors were upregulated in biofilm stages. Stress response factors are major groups enabling the biofilm cells to resist the pH stress, oxidative stress and nutrient deprivation. Proteome analysis also elucidated interesting observations on the relevance of *luxS* mediated quorum sensing and pheromone associated proteins in biofilm development of fsr negative non-gelatinase producing Enterococci. The high-level expression of rhamnose biosynthesis genes proposes the role of rhamnopolysaccharide as a major component strengthening the biofilm EPS matrix. In the present study, all the major pathways found to be associated with the biofilm phenotype might be playing an inevitable role in biofilm associated infections. Indeed, the generated proteome data had provided a set of interesting targets in the biofilm forming mechanism of *E. faecalis*. Future gene mutation studies are warranted to prove the exact role of these factors in biofilm establishment. The identified targets along with the advanced computational drug discovery approaches and high-throughput screening would help to accelerate the development of suitable anti-biofilm agents against Enterococcal biofilm.

## Methods

### Bacterial strain and growth condition

*E. faecalis* SK460, isolated from chronic diabetic ulcer patient was used for quantitative proteomics approach to identify biofilm-associated proteins. The study was approved by the Institutional Human Ethics Committee (Reference number RGCB-IEC No. IHEC/01/2013/11) of Rajiv Gandhi Centre for Biotechnology, Kerala, India. The draft genome sequence of this strain was deposited at NCBI Genbank with accession no. NIXL00000000 [41]. The strain was cultured in Luria Bertani broth (Becton Dickinson, New Jersey, United States) at 37 °C with 150 rpm. The media used for culturing biofilm was Tryptic soy broth (Himedia, Mumbai, India) with 0.75% Glucose.

### Biofilm quantification and imaging

For confocal imaging, an overnight culture of *E. faecalis* SK460 was diluted to OD600 = 0.1 and 4 ml of diluted broth was distributed to 6 well microtitre plate (Nunc, Roskilde, Denmark). A sterile square glass coverslip was inserted horizontally in each well and incubated at 37 °C in a static condition for two-time intervals (12 h, 24 h). Biofilm staining was carried out with Syto9 (Invitrogen, California, United States) as described previously [42]. The slides were then observed using a Nikon Eclipse Ti Confocal Laser scanning inverted microscope (Nikon Instruments Corporation, New York, United States). The measurement of biofilm thickness was performed using NIS-Element AR software, version 4.00.04.

### Cell extract preparation

The strain was cultured in different planktonic and biofilm stages to carry out quantitative proteome analysis. The test strain was inoculated in Tryptic soy broth (with 0.75% glucose) and incubated at 37 °C with shaking at 180 rpm. After overnight incubation, the culture was diluted to adjust the optical density to 0.1 at 600 nm.

### Harvesting planktonic stage cells

One hundred milliliter of diluted culture was dispensed each in 250 ml conical flasks for two different time points (12 h, 24 h) in triplicates and incubated at 37 °C with shaking at 150 rpm. After proper incubation, the cells were pelleted and washed twice with sterile ice-cold phosphate buffered saline (PBS, pH 7.4) and stored at -80 °C till protein isolation.

### Harvesting biofilm stage cells

Similarly, the diluted culture was dispensed to Cell Culture Treated EasYFlasks, 175 cm^2^ (Nunc, Roskilde, Denmark) at two-time points (12 h, 24 h) in triplicates and incubated at 37 °C in a static condition. After incubation, the used media along with the planktonic cells were washed off and the flask was rinsed twice with sterile PBS to remove the settled planktonic cells. The biofilm cells were then scraped off, pooled in sterile ice-cold PBS, pelleted and stored at -80 °C till protein isolation.

### Protein sample preparation

Protein isolation from cell pellets of different planktonic and biofilm stages was carried out by bead beating as described previously [43]. Briefly, cell pellets were resuspended in PBS (1 ml PBS per gram of cells) and transferred to 2 ml microcentrifuge tubes containing glass beads (0.5 mm) and protease inhibitor (1 mM Phenylmethyl Sulfonyl Fluoride, PMSF) and incubated on ice for 5 min. The tubes were then placed in a Mini Bead beater (BioSpec Products, USA) and subjected to one-minute pulses at 4200 rpm for three times with 1 min interval. The suspensions were centrifuged at 13,000 rpm for 10 min at 4 °C and the supernatant was collected. Protein concentration was estimated by bicinchoninic acid protein assay kit as followed by the manufacturer’s instructions (Thermo Scientific, Massachusetts, United States). Three biological replicates and three technical replicates were used in this experiment. 100 μg of protein (1 μg/μl) from each sample was made up to 100 μl with 50 mM Ammonium bicarbonate and subjected to disulfide reduction, alkylation and in-solution tryptic digestion as followed by previous publication [44]. The digested peptide solution was centrifuged at maximum rpm at 4 °C for 12 min and the supernatant was stored at −20°C until the analysis using Liquid Chromatography-Tandem Mass Spectrometry (LC-MS/MS).

### LC-MS/MS analysis

Peptide samples were analyzed by nano-LC/MS_E_ (at elevated energy) using a nanoACQUITY UPLC® system (Waters, Altrincham, UK) coupled to a Quadrupole-Time of Flight (Q/TOF) mass spectrometer (SYNAPT-G2, Waters, Altrincham, UK). Instrument operation and control for both the system was done employing MassLynx4.1 SCN781 software. The peptides were separated by reverse-phase column chromatography in the nanoACQUITY UPLC®. Peptides eluted from the nano-LC were subjected to mass spectrometric analysis on a SYNAPT® G2 High Definition MS™ System (Waters, Altrincham, UK). All analyses were performed using positive mode ESI using a NanoLockSpray™source. The detailed operational set up was followed as in previous publications [44].

### Data analysis

ProteinLynx Global SERVER™ (PLGS) v2.5.3 (Waters, Altrincham, UK) was used to analyze the acquired ion mobility enhanced MS^E^ spectra for protein identification as well as for the label-free relative protein quantification. Data processing and parameter setups were carried out as followed by [44]. The sequence database of the reference strain *E. faecalis* V583 (NCBI Reference Sequence: NC_004668.1) was used for database search. The false positive rate (FPR) was set to 4% with a randomized database, appended to the original one. The parameters for protein identification were made in such a way that a peptide was required to have at least one fragment ion match, a protein was required to have at least three fragment ion matches, and two peptide matches for identification. The peptides with 50% or more probability to be present in the mixture and detected with a score above 20, as calculated by the software were selected for proteomic analysis [45]. Data sets were normalized using the ‘internal standard-normalization’ function of PLGS and label-free quantitative analysis was performed by comparing the normalized peak area/intensity of identified peptides between the samples.

### Analysis of physicochemical properties of identified proteome

The hydrophobicity of the identified proteins was calculated based on grand average hydropathy (GRAVY) value using the online GRAVY calculator (http://www.gravy-calculator.de/). The GRAVY score was calculated by dividing the sum of hydropathy values of all the amino acids by the protein length [46]. Isoelectric point (pI) and Molecular weight (MW) of the proteins was calculated using compute pI/MW tool of ExPASy Bioinformatics Resource Portal (https://web.expasy.org/compute_pi/).

### Gene ontology analysis

The total proteins from all the stages were analyzed using the Venny tool 2.1.0 to identify proteins specific to the planktonic and biofilm stages. The total proteome of the planktonic stage was obtained by compiling the total proteins identified in the triplicates of two different time points of planktonic stage viz., 12 h and 24 h. Similarly, the total proteome of biofilm stages was compiled. The identified proteins were subjected to Gene Ontology (GO) and Kyoto Encyclopedia of Gene and Genomes (KEGG) analysis using Database for Annotation, Visualization and Integrated Discovery (DAVID) functional Annotation Bioinformatic analyzer [47, 48]. DAVID generates Expression Analysis Systematic Explorer (EASE) score, a modified Fisher’s Exact *P*-value for each term and the GO and KEGG pathway terms with a *P* value ≤0.1 was considered as enriched. Average relative expression values of at least two biological replicates were used and a fold change of more than 30% (ratio of either < 0.70 for downregulation and > 1.3 for upregulation) was considered to be significantly altered levels of expression. Protein-protein interaction networks of differentially overexpressed proteins in biofilm stages were generated using STRING 10.5 (https://string-db.org/) [49] and a confidence view was generated by setting the filter to medium confidence (0.400).

### Real-time PCR analysis

RNA was isolated from planktonic and biofilm stages of *E. faecalis* SK460 using RNeasy mini kit (Qiagen, Hilden, Germany) as per manufacturer’s protocol involving lysis with lysozyme and finally treated with DNase-1 (Sigma-Aldrich, Missouri, United States). Conventional PCR using 16SrRNA gene was performed to check the DNA contamination. cDNA was synthesized using Prime Script™ 1st strand cDNA synthesis kit according to the manufacturer’s protocol (Applied Biosystems, California, United States) and stored at ^−^ 20 °C.

Primers of selected genes were designed using PrimerQuest tool of Integrated DNA Technologies (Integrated DNA Technologies, Inc., California, US) and are shown in Additional file 1: Table S1. Power SYBR Green PCR Master Mix (Takara, Kusatsu, Japan) was used for real-time PCR in Applied Biosystems 7900HT Fast Real-Time PCR System as per the manufacturer’s suggestion. The reaction procedure was as follows: incubation at 95 °C for 10 min followed by 40 cycles of 95 °C for 10 s, 49 °C for 15 s and 60 °C for 45 s. Three independent reactions were conducted with triplicates for each gene and reaction mixture without RNA template was used as negative control for each set of primers. Expression analysis of each gene was calculated based on at least two independent experiments. Melting curve analysis was done to verify primer specificity and relative expression values were calculated as 2-∆(CT Target - CT reference) with 23S rRNA gene as housekeeping gene.

## Additional files

Additional file 1: **Table S1.** List of primers used for qRT-PCR (DOC 35 kb)

Additional file 2: **Figure S1.** a) Scatter plot showing the distribution of GRAVY index for the proteins identified in planktonic and biofilm stages. b). Distribution of the predicted molecular weight (MW) and isoelectric point (pI) for the total proteins obtained in planktonic and biofilm stages. (TIF 8548 kb)

Additional file 3: **Figure S2.** Functional interactome of up-regulated proteins in biofilm stages, generated using STRING network prediction algorithm (Version 10.5). Colored nodes: query proteins and first shell of interactors. Empty nodes: proteins of unknown 3D structure. Filled nodes: some 3D structure is known or predicted. Edges represent protein-protein associations. Known interactions- Pink represents experimentally determined, wathet-blue represents from curated database, predicted interactions -green represents gene neighborhood, dark blue represents gene co-occurrence, red represents gene fusions, and black represents co-expression. (TIF 8948 kb)

## Data Availability

The mass spectrometry proteomics data have been deposited to the ProteomeXchange Consortium via the PRIDE partner repository with the dataset identifier PXD010770.

## Abbreviations

DAVID: Database for Annotation, Visualization and Integrated Discovery
EASE: Expression Analysis Systematic Explorer
EPS: Extra polymeric substance
FPR: False positive rate
fsr: Fecal Streptococci regulator
GO: Gene Ontology
KEGG: Kyoto Encyclopedia of Gene and Genomes
LC-MS/MS: Liquid Chromatography - Tandem Mass Spectrometry
LuxS: S-ribosylhomocysteinase
PBS: Phosphate buffered saline
PLGS: ProteinLynx Global SERVER™
