# Supplementary material for: Decoding the proteomic changes involved in the biofilm formation of Enterococcus faecalis SK460 to elucidate potential biofilm determinants
Source: BMC Microbiol. 2019 Jun 28;19:146. doi: 10.1186/s12866-019-1527-2 (PMC6599329; doi:10.1186/s12866-019-1527-2)
Supplement: Supplementary file 1 — Table S1. List of primers used for qRT-PCR (DOC 35 kb) [file 12866_2019_1527_MOESM1_ESM.doc]

**Decoding the proteomic changes involved in the biofilm formation of *Enterococcus faecalis* SK460 to elucidate potential biofilm determinants**

Additional file 1

**Table S1.** List of primers used for qRT-PCR

| **Target gene** | **Primer** | **Oligonucleotide sequence (5'−3')** |
| --- | --- | --- |
| *E. faecalis* 23S rRNA | **EF23SF** | CCTATCGGCCTCGGCTTAG |
|  | **EF23SR** | AGCGAAAGACAGGTGAGAATCC |
| dTDP-glucose 4,6-dehydratase | **rfbB F** | GGACTTGAAGGATTGTAAG |
|  | **rfbB R** | CATGTATCGACTGATGAAG |
| S-ribosylhomocysteinase | **luxS F** | ATGTTGATCCCACATAATC |
|  | **luxS R** | CACGTTGGAACATTTATTAG |
| Fibronectin/fibrinogen binding proteins | **Fbn F** | GGTCTTTCTCTGCTTTAC |
|  | **Fbn R** | TACGCCCATTCCTTATC |
| Arginine deiminase | **ArcA F** | TATCTGGAGAGACTGTTG |
|  | **ArcA R** | GCAGCTAAGTCCTCTAA |
| Pheromone cAD1 lipoprotein | **cAD1 F** | CGGTGTGGAAGTAGTAA |
|  | **cAD1 R** | GATTGTGTCTGTGTTACC |
