# Supplementary figures and images for: Decoding the proteomic changes involved in the biofilm formation of Enterococcus faecalis SK460 to elucidate potential biofilm determinants
Source: BMC Microbiol. 2019 Jun 28;19:146. doi: 10.1186/s12866-019-1527-2 (PMC6599329; doi:10.1186/s12866-019-1527-2)

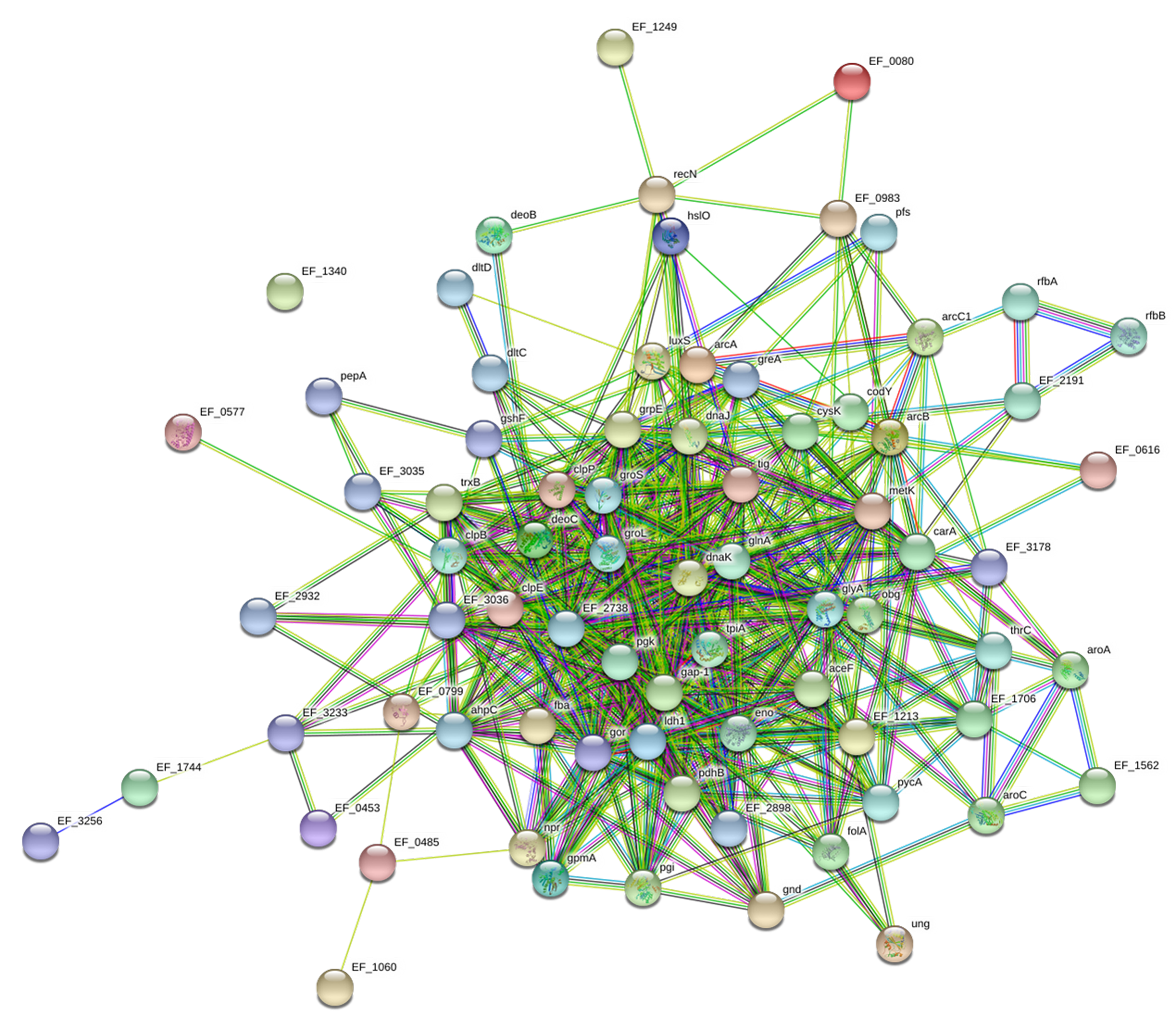

Supplement: Supplementary file 3 — Figure S2. Functional interactome of up-regulated proteins in biofilm stages, generated using STRING network prediction algorithm (Version 10.5). Colored nodes: query proteins and first shell of interactors. Empty nodes: proteins of unknown 3D structure. Filled nodes: some 3D structure is known or predicted. Edges represent protein-protein associations. Known interactions- Pink represents experimentally determined, wathet-blue represents from curated database, predicted interactions -green represents gene neighborhood, dark blue represents gene co-occurrence, red represents gene fusions, and black represents co-expression. (TIF 8948 kb) [file 12866_2019_1527_MOESM3_ESM.tif]
